# The Role of Nitric Oxide in the Micro- and Macrovascular Response to a 7-Day High-Salt Diet in Healthy Individuals

**DOI:** 10.3390/ijms24087157

**Published:** 2023-04-12

**Authors:** Ivana Tolj, Ana Stupin, Ines Drenjančević, Petar Šušnjara, Leon Perić, Marko Stupin

**Affiliations:** 1 Department of Internal Medicine and History of Medicine, Faculty of Medicine Osijek, Josip Juraj University of Osijek, J. Huttlera 4, 31000 Osijek, Croatia; ivanatolj5@gmail.com; 2 Department of Nephrology, University Hospital Osijek, J. Huttlera 4, 31000 Osijek, Croatia; 3 Department of Physiology and Immunology, Faculty of Medicine Osijek, Josip Juraj Strossmayer University of Osijek, J. Huttlera 4, 31000 Osijek, Croatia; ines.drenjancevic@mefos.hr (I.D.); psusnjara@mefos.hr (P.Š.); 4 Scientific Center of Excellence for Personalized Health Care, Josip Juraj Strossmayer University of Osijek, Trg Svetog Trojstva 3, 31000 Osijek, Croatia; 5 Department of Emergency Medicine of Osijek-Baranja County, J. Huttlera 2, 31000 Osijek, Croatia; lperic1@yahoo.com; 6 Department for Cardiovascular Disease, University Hospital Osijek, J. Huttlera 4, 31000 Osijek, Croatia

**Keywords:** high-salt diet, endothelium, nitric oxide, nitric oxide synthase, vascular endothelial growth, microcirculation, macrocirculation

## Abstract

This study aimed to investigate the specific role of nitric oxide (NO) in micro- and macrovascular response to a 7-day high-salt (HS) diet, specifically by measuring skin microvascular local thermal hyperemia and the flow-mediated dilation of the brachial artery, as well as serum NO and three NO synthase enzyme (NOS) isoform concentrations in healthy individuals. It also aimed to examine the concept of non-osmotic sodium storage in the skin following the HS diet by measuring body fluid status and systemic hemodynamic responses, as well as serum vascular endothelial growth factor C (VEGF-C) concentration. Forty-six young, healthy individuals completed a 7-day low-salt diet, followed by a 7-day HS diet protocol. The 7-day HS diet resulted in impaired NO-mediated endothelial vasodilation in peripheral microcirculation and conduit arteries, in increased eNOS, decreased nNOS, and unchanged iNOS concentration and NO serum level. The HS diet did not change the volume of interstitial fluid, the systemic vascular resistance or the VEGF-C serum level. These results indicate that the 7-day HS-diet induces systemic impairment of NO-mediated endothelial vasodilation, while dissociation in the eNOS and nNOS response indicates complex adaptation of main NO-generating enzyme isoforms to HS intake in healthy individuals. Our results failed to support the concept of non-osmotic sodium storage.

## 1. Introduction

According to a systematic analysis of risk factors that contribute to disease and disability/disease-adjusted life years, arterial hypertension (AH) is the major preventable cause of morbidity and mortality globally [1], including Croatia. The results obtained from the scientific research project Epidemiology of hypertension in Croatia (EH-UH 1), which was completed 20 years ago, reported a prevalence of AH of 37% [2], with very poor treatment control—about 20%. One of the main reasons for the high prevalence and poor AH control is the excessive intake of table salt, which in Croatia amounts to more than 11 g per day (average 11.6 g of salt/day; 13.3 g/day for men and 10.2 g/day for women). This data was the basis for the development of the Strategic Plan for Reduction of Salt Intake in Croatia, aiming to reduce daily salt intake to 9.3 g in 2019 [3]. Available data from 2019 show that average daily salt intake has decreased by 1.6 g of salt per day (1.9 g of salt/day in men and 1.0 g of salt per day in women), which indicates the necessity for further public health programs in order to bring the salt intake in Croatia closer to the values recommended by the World Health Organization (5 g of salt/day) [4]. 

Alongside the clear association between high-salt (HS) intake and the development and progression of AH, some evidence indicates that increased salt intake affects vascular and endothelial function, even in the absence of changes in arterial blood pressure (ABP) [2,5,6] or body composition (fluid) changes in healthy individuals [7,8,9,10]. Endothelial dysfunction (ED) is a precursor and the earliest observable outcome of cardiovascular diseases (CVDs), characterized by inappropriate activation of the endothelium [5,11]. ED has been demonstrated at macro- and microcirculatory level. A short-term high-salt (HS) diet (one HS meal or a 7-day HS diet) has been shown to reduce brachial artery flow-mediated dilation (FMD) independently of ABP changes, in healthy subjects [12,13,14,15,16]. As one of the most important functions of the endothelium is to release vasoactive substances and thereby regulate vascular blood flow, ED commonly refers to reduced production and/or bioavailability of the main endothelial vasodilator nitric oxide (NO), and/or an imbalance in the contribution of vasodilator (NO, prostacyclin, and other endothelial hyperpolarization factors) and vasoconstrictor metabolites (endothelin 1, thromboxane, 20-hydroxyeicosatetraenoic acid etc.) derived from the endothelium, which results in impaired vascular relaxation mechanisms [17,18].

Excessive salt intake may impair endothelial vascular function at both micro- and macrocirculation levels, potentially by suppression of the renin-angiotensin system (RAS) activity, an increased level of oxidative stress, and the activation of the endothelium, resulting in endothelial–leukocyte interaction and modulation of the inflammatory response, in otherwise healthy individuals [19]. The underlying mechanisms mediating these changes during salt loading include the production of endothelial vasoconstrictor metabolites (e.g., arachidonic acid metabolites derived from cyclooxygenases). An earlier study by our research group showed that short-term exposure to an HS diet induces an increase in the serum concentration of thromboxane (TXA2), a vasoconstrictor derived from cyclooxygenase enzymes (COX-1 and -2), but also that a change in the concentration of other vasoconstrictor metabolites of the cyclooxygenase enzymes (specifically COX-1) can play an important role in the development of microvascular dysfunction in healthy individuals [8]. 

Besides the increased production or sensitivity to vasoconstrictor metabolites, reduced bioavailability of the main endothelial vasodilation factor NO plays an important role in the development of ED, due to excessive salt intake, as suggested in animal models [19]. Moreover, it has been shown that reduction in the bioavailability of NO is associated with increased levels of oxidative stress, which leads to the impairment of endothelium-dependent vasodilation in rats fed a high-salt diet [19]. In particular, the increased level of superoxide ion (O_2_−•) reacts with NO and results in the formation of peroxynitrite (ONOO-) [20], and/or it may affect the activity of the enzyme that generates endothelial NO, i.e., endothelial NO synthase (eNOS) [21]. Interestingly, only a few studies have investigated this effect in humans; Greaney et al. showed that 7 days of an HS diet caused impairment of microvascular reactivity in forearm skin in response to local skin heating in twelve normotensive adults without ABP changes [7]. In the same study, it was shown that the local application of ascorbic acid alleviates or reverses damage to the microvascular function [7]. A study by our research group demonstrated that vasodilation of arterioles isolated from gluteal subcutaneous adipose tissue in response to acetylcholine (ACh) or flow changes was no longer mediated predominantly by NO, but that other vasodilator metabolites originating from COX and cytochromes P450 (CYP450) took part in this vasodilation following a 7-day HS diet [9]. For the first time, the same group investigated the effect of an HS diet on measurable biomarkers of oxidative stress in healthy subjects, and showed that HS loading results in a decrease in antioxidant capacity and an increase in the level of oxidative stress [22]. In addition, enhanced antioxidant defense by the supplementation of vitamins C and E during the HS diet reversed these changes [22]. However, there is still a lack of studies that investigate the specific effect of short-term HS intake specifically on NO and NO-mediated endothelium-dependent dilation in both micro and macrocirculation, using both the biochemical measurements of NO bioavailability and the activity of enzymes involved in its synthesis, as well as functional measurements of NO-dependent dilatation in different vascular beds in healthy individuals.

It is worth noting that salt-induced AH can develop in salt-sensitive rats without increasing the volume of body fluids, which in recent years was the basis for the introduction of the concept of reversible, non-osmotic, extra-renal sodium storage at the tissue level [23]. The main reservoir of inactive sodium would be the subcutaneous interstitium, but also bone, cartilage, and skeletal muscles. Large negatively charged polymers, glycosaminoglycans (GAGs), have been proposed as a base for sodium binding and storage at various tissues [24]. Further studies identified elements of the regulatory cascade of sodium storage as follows; negatively charged GAGs bind sodium and create a hyperosmotic environment; increased tissue tone induces invasion and activation of monocytes/macrophages, and such activated cells express the enhancer of tonicity-responsive protein binding (TonEBP) that stimulates the secretion of the vascular endothelial growth factor C (VEGF-C). VEGF-C, through the vascular endothelial growth factor receptor-3 (VEGFR-3) activation, stimulates lymphangiogenesis and the capacity of the lymphatic system to adapt, as well as to return sodium and accumulated volume from the interstitium back into the circulation [24,25]. Moreover, there are indications that VEGF-C enhances the expression and activity of eNOS, leading to a decrease in vascular resistance [25]. So far, the effectiveness of this buffer system has been demonstrated only in animal models. 

Thus, the present study aimed to investigate the effect of a 7-day HS diet on the specific role of nitric oxide in the micro- and macrovascular response to a 7-day salt load in healthy individuals, by measuring the endothelium-dependent nitric oxide (NO) mediated vasodilation of micro- and macrocirculation, serum levels of NO, and protein levels of three isoforms of the nitric oxide synthase enzyme (NOS), which are involved in NO production. An additional aim was to examine the concept of non-osmotic sodium storage in the skin by measuring the interstitial fluid volume, VEGF-C serum levels and potential effects of the HS diet on interstitial fluid volume and peripheral vascular resistance in young healthy individuals. 

## 2. Results

Forty-six healthy, lean, normotensive individuals of both sexes, of age ranging between 18 and 24 years, completed a two-week dietary salt perturbation protocol involving a 7-day low-salt (LS) diet, which included a wash-out period for the normalization of the daily salt intake of all participants, followed by a 7-day HS diet. The HS diet did not induce any significant change in the subjects’ body mass index (BMI) or waist circumference, nor in body composition and fluid status, compared to the LS diet conditions. This information is presented in Table 1. 

All participants had normal renal function and serum electrolytes, and did not have any active inflammatory process at the moment of entering the study protocol. The HS diet did not affect renal function (urea and creatinine), serum sodium and potassium, or the high-sensitivity C-reactive protein (hsCRP) level, compared to the LS diet conditions. The HS diet significantly decreased the serum calcium concentration, compared to the LS conditions; nevertheless, the values for both the LS and HS diet conditions were within the normal reference range (calcium 2.14–2.53 mmol/L). A significant increase in 24-h sodium excretion, as well as in calculated daily salt intake, confirmed that participants conformed to the given dietary protocols. The 24-h urine volume significantly increased following the HS diet, while the 24-h urine creatinine coefficient, urea, protein, albumin and potassium excretion were not significantly affected by the HS diet compared to the LS diet condition. Changes in standard biochemical venous blood and 24-h urine parameters following the HS diet are summarized in Table 2.

### 2.1. Arterial Blood Pressure and Systemic Hemodynamic

ABP and systemic hemodynamic responses to the HS diet are presented in Table 3. As previously reported, the HS diet did not induce significant change in systolic blood pressure (SBP), diastolic blood pressure (DBP) or mean arterial blood pressure (MABP), compared to LS conditions. Moreover, since a significant increase in daily salt intake and 24-h sodium excretion was not accompanied by changes in the ABP level, all the observed changes could be considered as independent of ABP. 

Heart rate was not significantly changed following the HS diet compared to LS conditions. In addition, stroke volume, cardiac output, cardiac index, systemic vascular resistance index (SVRI) and total arterial compliance index were not significantly changed after the HS diet, compared to LS conditions. 

### 2.2. Micro- and Macrovascular NO-Dependent Endothelial Vasodilation

**Figure 1:**
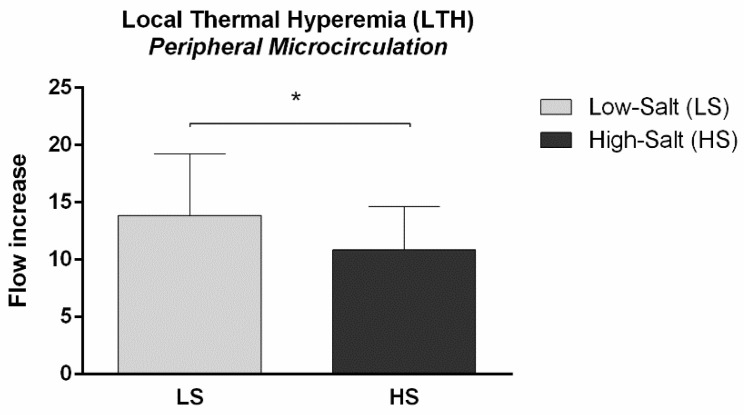
The effect of a 7-day high-salt (HS) diet on a microvascular nitric oxide (NO)-mediated vasodilation in healthy individuals. Local thermal hyperemia (LTH) of forearm skin microcirculation measured by laser Doppler flowmetry (LDF) significantly decreased following 7-day HS diet compared to LS diet condition in healthy individuals. * *p* = 0.009, LS vs. HS (Paired *t*-test).

Microvascular NO-dependent endothelial vasodilation assessed by microvascular local thermal hyperemia of the forearm skin was significantly decreased following the HS diet, compared to LS conditions (local thermal hyperemia (LTH) flow increase LS 13.9 ± 5.4 vs. HS 10.9 ± 3.8, *p* = 0.009) (Figure 1). 

**Figure 2:**
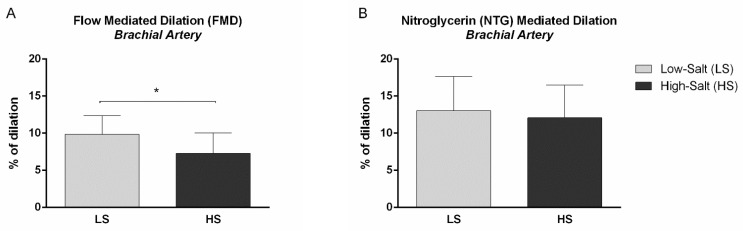
The effect of a 7-day high-salt (HS) diet on a macrovascular endothelial-dependent (**A**) and -independent (**B**) vasodilation in healthy individuals. (**A**) Flow-induced dilation (FMD) of brachial artery significantly decreased following 7-day HS diet, compared to LS diet condition in healthy individuals. * *p* < 0.001, LS vs. HS (Paired *t*-test). (**B**) Nitroglycerin (NTG)-mediated dilation of brachial artery remained unchanged following 7-day HS diet, compared to LS conditions in healthy individuals.

Furthermore, NO-dependent macrovascular endothelial vasodilation assessed by flow-mediated dilation of the brachial artery was significantly decreased following the HS diet, compared to LS conditions (FMD % of dilation LS 9.9 ± 2.5 vs. HS 7.3 ± 2.8, *p* < 0.001) (Figure 2A). On the other hand, no significant change was observed in endothelium-independent macrovascular dilation assessed by the nitroglycerin (NTG)-mediated dilation of the brachial artery following the HS diet, compared to LS (NTG-mediated dilation % of dilation LS 13.0 ± 4.6 vs. HS 12.1 ± 4.4, *p* = 0.157) (Figure 2B).

### 2.3. Nitric Oxide, Nitric Oxide Synthases and Vascular Endothelial Growth Factor C Serum Levels

**Figure 3:**
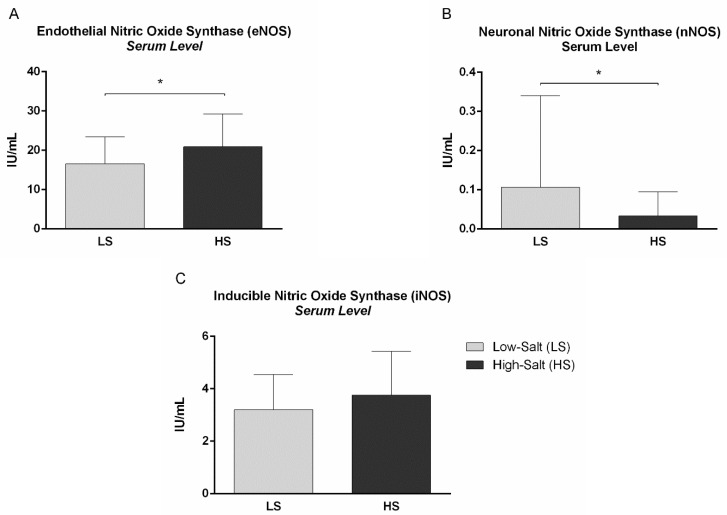
The effect of a 7-day high-salt (HS) diet on endothelial (eNOS) (**A**), neuronal (nNOS) (**B**) and inducible nitric oxide synthase (iNOS) (**C**) serum level in healthy individuals. (**A**) eNOS serum level significantly increased following 7-day HS diet, compared to LS diet condition in healthy individuals. * *p* = 0.039, LS vs. HS (Paired *t*-test). (**B**) nNOS level significantly decreased following 7-day HS diet, compared to LS diet condition in healthy individuals. * *p* = 0.030, LS vs. HS (Paired *t*-test). (**C**) iNOS serum level remained unchanged following HS diet, compared to LS conditions in healthy individuals.

Effects of a 7-day HS diet on NO and VEGF-C serum levels are presented in Table 4, while the effects of HS loading on NOS level are presented in Figure 3. No significant change was observed in VEGF-C serum level following the HS diet, compared to LS conditions. The endothelial NOS (eNOS) serum level was significantly increased (Figure 3A), while the neuronal NOS (nNOS) level was significantly decreased following the HS diet, compared to LS conditions in healthy individuals (Figure 3B). The NO, as well as the inducible NOS (iNOS) serum level, did not significantly change following the HS diet, compared to LS (Figure 3C). 

### 2.4. Correlations

Correlations between 24-h sodium excretion / NO serum concentration and endothelial vascular response/NOS isoforms serum concentrations are presented in Table 5. There was a weakly negative correlation between the 24-h sodium excretion and the LTH response of skin microcirculation (R = −0.254, *p* = 0.050), as well as a moderately negative correlation between the 24-h sodium excretion and the FMD of the brachial artery (R = −0.322, *p* = 0.015). There was also a moderately negative correlation between the 24-h sodium excretion and the nNOS serum level (R = −0.483, *p* = 0.002), while 24-h sodium excretion was not associated with eNOS or iNOS serum concentration. Interestingly, the serum NO concentration moderately positively correlated with the LTH response of skin microcirculation (R = 0.555, *p* = 0.017).

SVRI and VEGF-C serum level did not show a significant mutual association, nor an individual association with 24-h sodium excretion, with the serum level of NOS isoforms or with the functional indicators of endothelium-dependent vasodilation in micro- and macrocirculation.

## 3. Discussion

The main finding of the present study was that a 7-day HS diet in healthy individuals impaired the NO-mediated endothelial vasodilation in both the peripheral microcirculation and the conduit arteries of healthy individuals, independent of ABP changes. Moreover, 24-h sodium excretion was negatively associated with both local microvascular LTH and FMD of brachial arteries. There was a positive correlation between NO serum level and LTH response of skin microcirculation, which confirmed the strong dependence of the microvascular response to these stimuli on the main endothelial vasodilator, NO. Interestingly, the serum level of eNOS was significantly increased while the nNOS level was decreased following the HS diet, indicating a complex adaptation of the main NO-generating enzyme isoforms to the HS intake in healthy individuals. However, regarding indirect assessment of the concept of non-osmotic sodium storage in the skin performed in the present study, we failed to demonstrate a significant influence of HS loading on potential determinants of such concept, in particular the serum VEGF-C level and systemic vascular resistance index in healthy individuals. The present results lead to the conclusion that at least such a short-term dietary salt modulation does not induce significant changes on the suggested axis which, via VEGF-C and the stimulation of lymphangiogenesis (and potentially eNOS), reduces vascular resistance and, consequently, affects ABP in terms of HS intake.

Studies in animal models consistently indicate that HS dietary intake can lead to a widespread ED in different vascular beds, but such effect was shown to be more variable in large conductive vessels than in smaller blood vessels (resistance arteries) [26,27,28]. In animals, HS intake (NaCl chow content between 4% and 8% by weight, lasting from 3 days up to 8 weeks) results in impaired endothelium-dependent vasodilation in resistance arteries (i.e., skeletal muscle, cerebral, mesenteric), characterized by a profound reduction in NO bioavailability, and independent of any increases in ABP [19,29,30]. There is mounting evidence to suggest that increased level of oxidative stress plays a central role in the impairment of endothelial-dependent vasodilation in rats fed an HS diet [19]. Moreover, the increased level of O_2_−• reduces NO bioavailability, due to oxidation of NO and formation of ONOO-, or by affecting eNOS activity [20]. 

The late hyperemic plateau following local skin heating measured by laser Doppler flowmetry (LDF) depends predominantly on endothelial NO [31], and serves as an excellent model for assessment of microvascular endothelium-dependent, NO-mediated vasodilation. The present study, for the first time, demonstrated a uniformly impaired NO-dependent endothelial response in both skin microcirculation and the brachial artery, which was independent of ABP changes in healthy, young individuals. Present results are in line with results from Greaney and al., which demonstrated that a 7-day HS intake reduced red blood cell flux during local heating of forearm microcirculation in 12 normotensive adults, also in the absence of ABP changes [7]. Similarly, FMD of brachial artery declines in response to dietary salt loading in men and women, as well as in both salt-sensitive and salt-resistant adults, supporting the thesis that HS loading has an adverse effect on vascular (endothelial) function that is independent of ABP changes [9,32,33,34]. This deleterious effect of HS intake on an NO-dependent vasodilation of the brachial artery was confirmed by the results of the present study, which was performed on a larger sample of healthy individuals of both sexes. Taken together, there is strong evidence that a 7-day HS diet in healthy individuals induces functional vascular impairment by affecting the endothelium, and in particular the endothelial response which is dependent on its main vasodilator, NO. Moreover, our previous report, showing that enhanced antioxidant defense by vitamin C and vitamin E administration during HS loading reverses changes in oxidative status and prevents impairment of the peripheral microvascular endothelial function [22], is in line with the abovementioned animal studies, and potentiates the pivotal role of increased oxidative stress in reducing the bioavailability of NO, resulting in impaired endothelial function during HS loading. 

The present study demonstrated an increased serum concentration of eNOS, a decreased concentration of nNOS, and an unchanged concentration of iNOS following HS intake. eNOS is mostly expressed in endothelial cells, and appears to be a homeostatic regulator of numerous essential cardiovascular functions, e.g., vasodilation, inhibition of platelet aggregation and adhesion, inhibition of leucocyte adhesion and vascular inflammation, control of vascular smooth muscle proliferation, stimulation of angiogenesis, and activation of endothelial progenitor cells [35]. The conventional notion, that eNOS is mostly responsible for the regulation of vascular tone in the periphery, has been challenged by a human study which indicated that nNOS too may play an important role in the regulation of vascular tone, independent of its effects on the central nervous system. Interestingly, a selective nNOS inhibitor administration (S-methyl-L-thiocitrulline (SMTC)) was shown to reduce basal blood flow in the human forearm and in the coronary circulation, without affecting classical eNOS-mediated vasodilatation in response to acetylcholine, or vascular shear stress [36]. Indeed, in recent years, an increasing number of reports have confirmed the significance of nNOS in the periphery, where many smooth muscle tissues are innervated by nitrergic nerves containing nNOS, thus generating NO [35]. iNOS is not usually expressed in cells, but its expression can be induced by various stimuli such as bacterial lipopolysaccharide, cytokines, and other agents (e.g., in macrophages, tumor cells, hepatocytes, etc.) [35]. The unchanged iNOS concentration, together with the unaltered hsCRP concentration following the 7-day HS diet, is in line with the earlier findings of our research group that a short-term HS diet (with the same dietary protocol as in the present study) induces an early pro-inflammatory, but also a counterbalancing anti-inflammatory response, which may protect healthy salt-resistant individuals from more prominent endothelial activation and dysfunction [37]. 

Interestingly, despite preserved levels of NO, NO-dependent vasodilation was attenuated after the HS diet intake. This may suggest that NO bioavailability or sensitivity to NO (via cellular down-signaling pathways) may have been affected by the HS diet. Decreased bioavailability of NO in CVDs has been mainly attributed to the increased oxidative stress, which results in an increased degradation of NO by its reaction with O_2_−•, but also in the conversion of eNOS from an NO-producing enzyme to an enzyme that generates O_2_−•, the process known as NOS uncoupling [35]. eNOS uncoupling implicates various mechanisms, including the oxidation of the cofactor BH4, depletion of L-arginine, accumulation of endogenous methylarginines, and S-glutathionylation of eNOS, etc. It is noteworthy that several studies have shown that cardiovascular risk factors are associated with an increase, rather than a decrease, in eNOS expression [38]. It has been demonstrated that the dismutation product of O_2_−•, hydrogen peroxide (H_2_O_2_), can increase eNOS expression through transcriptional and post-transcriptional mechanisms [39]. Thus, it seems than in terms of increased oxidative stress (also demonstrated due to a 7-day HS loading in healthy individuals) [22], an accelerated degradation of peripheral NO occurs, with NO and O_2_−• reacting to form ONOO-, which in turn diminishes levels of BH4, and leads to eNOS uncoupling and increased production of O_2_−• by the dysfunctional enzyme [35]. Such a vicious circle and transformation of eNOS from a protective enzyme to a contributor to oxidative stress has been observed in several in vitro models, in animal models of cardiovascular diseases, and in patients with cardiovascular risk factors [35]. For example, HS intake led to increased O_2_−• production and decreased NO bioavailability in rat aortas [40]. It has been demonstrated that uncoupled eNOS appears to be a major source of O_2_−• in spinotrapezius muscle arterioles of mice fed an HS diet [41]. Our earlier study in Sprague Dawley rats fed a 7-day HS diet reported reduced flow-induced dilation of isolated middle cerebral arteries, which was restored by the superoxide dismutase (SOD) mimetic, and reduced gene expression of iNOS, but not eNOS in isolated cerebral blood vessels [42]. However, the evidence on the effect of HS loading on NO level, as well as on NOS isoform expression/activity/level in human studies, is scarce. To our knowledge, this is the first study in healthy individuals reporting increased eNOS serum level, decreased serum nNOS level, a negative association of HS loading and nNOS, and decreased NO-mediated vasodilation following a 7-day HS diet, which, together with earlier demonstrated increased oxidative stress in salt-stressed individuals, is consistent with the reduced bioavailability of NO and eNOS uncoupling in individuals with high cardiovascular risk. Although there are limited indications that vascular smooth muscle cells also express low levels of nNOS, and have been shown to maintain some degree of vasodilation, especially when the predominant eNOS becomes dysfunctional, the finding of significantly decreased nNOS serum level in terms of increased (uncoupled) eNOS levels due to HS loading opens an intriguing research area on the specific roles of eNOS and nNOS in the physiological regulation of human vascular tone in vivo. 

A generally accepted view that the major factors in ABP elevation are inefficient renal sodium excretion and subsequent extracellular volume expansion has been challenged in the past few years; namely, the increasing number of reports showed the dissociation of sodium and volume homeostasis. For example, HS dietary intake may not cause extracellular volume expansion in human subjects [43] (also previously observed in our earlier, as well as in the present, study [10]), just as salt-induced hypertension may develop in salt-sensitive rats without body fluid expansion [44]. Thus, as the underlying mechanism, the concept of reversible, non-osmotic sodium storage at tissue levels has been introduced [24,45]. The major elements of such regulatory cascade of reversible sodium storage in the skin interstitium have been the negatively charged GAGs that bind sodium and create hypertonicity, which subsequently induces monocyte/macrophage invasion and activation expressing a TonEBP that stimulates VEGF-C secretion. VEGF-C, by increasing lymphangiogenesis, enhances the capacity of the lymphatic system to drain back accumulated sodium and volume from the interstitium back into circulation [24,45]. Moreover, it has been suggested that VEGF-C enhances the expression and activity of eNOS, which leads to the reduction in vascular resistance and ABP. The effectiveness of such a buffer system has been supported by studies in animal models. So far, limited evidence related to the concept of non-osmotic sodium deposition in a human model has shown that sodium storage in the skin increases with aging and that, depending on age, it is associated with reduced levels of VEGF-C in the circulation [46]. For example, the level of VEGF-C in patients with chronic kidney disease was higher in those on an HS diet, while there was a tendency to increase VEGF-C levels as well in healthy subjects on an HS diet, but this did not reach the level of statistical significance [47]. Another study demonstrated that inhibition of the VEGF-C signaling pathway in oncology patients increases arterial pressure values [48]. Interestingly, this discovery was confirmed in animal models, demonstrating that inhibition of angiogenesis causes salt accumulation in the interstitium and salt-sensitive hypertension [49]. The present study tested this concept by assessing several elements of proposed regulatory cascade, in particular the changes in extracellular water and interstitial fluid volume, serum level of VEGF-C, and systemic vascular resistance index following a 7-day HS diet in healthy individuals. Since we did not observe any significant effect in the share of extracellular water, volume of interstitial fluid, VEGF-C serum level, and SVRI in healthy individuals, our results failed to support the suggested concept, at least under the conditions of a 7-day salt load in young, healthy individuals. Nevertheless, the concept of the sodium buffer system in skin interstitium remains to be investigated in further studies in healthy individuals involving conditions of a longer period of salt loading or using different methodological approaches. 

Limitation of the study: Unchanged serum NO levels, despite evidently impaired NO-mediated vascular response in both micro- and macrocirculation, as well as changed serum eNOS and nNOS levels, could be potentially explained by methodological challenges in NO measurement. Furthermore, there are other reports that in many cases NO status in blood does not accurately reflect the corresponding NO status in tissues of interest [50]. On the other hand, the unchanged NO serum level and also unchanged total peripheral vascular resistance may indicate the preservation of NO generation that affects the basal flow. However, NO-mediated dilation seems to be inadequate in terms of the vascular challenge that requires a quick response of the vasculature and a high level of reactivity to a given stimulus.

In conclusion, the present study demonstrated that a 7-day HS diet impairs NO-mediated endothelial vasodilation in both microcirculation and conduit arteries of young, healthy, normotensive individuals of both sexes, independent of ABP changes, which was additionally confirmed by the weak-to-moderate negative association between 24-h sodium excretion and NO-mediated vascular responses. Such functional endothelial vascular impairment due to 7-day HS loading was accompanied by increased eNOS and decreased nNOS, supporting earlier reports on reduced bioavailability of NO and eNOS uncoupling under the condition of HS dietary intake. Moreover, unaltered total vascular peripheral resistance following HS loading suggests that early phases of endothelial activation/dysfunction could be characterized by the preserved basal flow NO responses, but could affect NO bioavailability or sensitivity to NO (via cellular down-signaling pathways). Furthermore, dissociation between eNOS and nNOS serum level in response to a 7-day HS diet opens a new research question on the specific roles of eNOS and nNOS in the physiological regulation of human vascular tone in vivo. The results on the unchanged share of extracellular water, volume of interstitial fluid, and SVRI in healthy individuals, do not support the proposed concept of non-osmotic sodium storage at tissue level, at least not in the conditions of such a short salt loading in healthy, young individuals. 

## 4. Materials and Methods

### 4.1. Study Population

Forty-six young, healthy women (N = 24) and men (N = 22), of age ranging between 18 and 29 years, participated in the present study. Participants were recruited by advertisement at the Faculty of Medicine Josip Juraj Strossmayer University of Osijek, Osijek, Croatia. The exclusion criteria for entering the study protocol were smoking, pregnancy, taking oral contraceptives, a significant change in body weight in the past year, hypertension or hypotension, coronary disease, diabetes, hyperlipidemia, kidney damage, cerebrovascular diseases or diseases of peripheral blood vessels, allergic/atopic diseases, recent (<1 month) infection, taking antibiotics and non-steroidal anti-inflammatory drugs for the past month, or taking any drugs that can have an effect on the endothelium. Each participant was informed about all the protocols and procedures included in the present study. In addition, each subject gave their informed consent before entering the study protocol. The study protocol was in accordance with the standards established by the last revision of the Declaration of Helsinki, and approved by the Ethics Committee of the Faculty of Medicine Osijek (Class: 602-04/21-08/07, Number: 2158-61-07-21-52). The study was performed in the Laboratory for Clinical and Sports Physiology, and the Laboratory for Molecular and Clinical Immunology of the Department of Physiology and Immunology at the Faculty of Medicine Osijek. This research is also a part of a clinical trial investigating effects of an HS diet on microvascular endothelial function in healthy individuals, registered at ClinicalTrials.gov (ID NCT02727426 Dietary Salt and Microvascular Function).

### 4.2. Study Design

The study was designed as a non-randomized controlled trial in which all participants were subjected to the same experimental protocol with two repeated measurements. The study protocol lasted for 14 days. In order to adjust basal dietary salt intake, all subjects were instructed to maintain the 7-day LS diet, which implied the intake of about 3.5 g of table salt per day, according to the DASH (Dietary Approaches to Stop Hypertension) eating plan (US Department of Health and Human Services, 2006). This “wash-out” period was followed by the 7 days of HS diet, which implied an intake of about 14.7 g of salt per day. In order for all subjects to take approximately the same amount of salt, the subjects continued to consume about 3.5 g of salt with food, while the rest of the salt was supplemented in the form of a commercially available kitchen-salt powder—11.2 g per day. Such a salt-load design is well established and previously described in the studies of our research group [8,10,22,37,51], as well as being aligned with other clinical studies that study the impact of a short-term HS diet on vascular function in the human population [19]. All procedures described below were performed in two repeated measurements for each subject, the first measurement following 7 days of the LS diet (LS condition), and the second measurement following 7 days of the HS diet (HS condition). Both study visits were organized in the morning, and all measurements were performed after overnight fasting. 

### 4.3. Weight Status, Body Composition and Body Fluid Status

To assess BMI, participants’ body height and body weight were measured. As the single best single indicator of other individual and multiple cardiovascular risk factors, waist circumference was measured. Subjects’ body composition and body fluid status were assessed using a 4-terminal non-invasive impedance analyzer (Maltron Bioscan 920-II, Maltron International Ltd., Rayleigh, UK), used to measure body composition and body fluid status. The manufacturer’s original software using empirically derived formulas generated data on the proportion of fat free mass (FFM%), fat mass (Fat%), total body water (TBW%), extracellular water (ECW%), intracellular water (ICW), plasma volume (PF), interstitial fluid volume (IF) and body density (kg/L).

### 4.4. Venous Blood and 24-h Urine Sampling and Analysis

A venous blood sample and a 24-h urine sample were taken from each participant under both LS and HS diet conditions. One part of the serum sample was immediately analyzed for electrolytes (sodium, potassium, calcium), urea and creatinine, and hsCRP, using standardized laboratory protocols and procedures. The other part of the serum sample was separated from venous blood, aliquoted, and stored in a refrigerator at −80 °C, until further experiments were performed.

The completeness of the 24-h urine collection was determined by an automatic laboratory program that, according to the default formula, calculates the creatinine coefficient from the creatinine value in urine, body weight and volume of 24-h urine. The 24-h urine excretion of Na, K, urea, creatinine, proteins and albumins was determined. Estimated daily salt intake in grams was calculated from the 24-h sodium excretion, using the appropriate formula [1-g salt (NaCl) = 393.4 mg Na = 17.1 mmol Na].

### 4.5. Systemic Hemodynamic and Arterial Blood Pressure

Systemic hemodynamic measurement was continuously monitored in each participant throughout 15 min while resting in a supine position. During that time period, at least three measurements of arterial blood pressure were performed. The final result of these measurements was the average value of all measured parameters in the specified time period. 

Systemic hemodynamics were assessed using an impedance cardiography (ICG) device (CardioScreen 2000 Professional, medis. Medizinische Messtechnik GmbH, Ilmenau, Germany), which is a non-invasive technology measuring total electrical conductivity of the thorax and its changes in time to process continuously a number of cardiodynamic parameters. ICG was performed using four electrical sensors attached to the patient’s neck and thorax which enable detection of the impedance changes caused by the blood flow and the liquid volume change in the thorax. Changes in the electrical potential of the thorax were also recorded, providing a signal similar to an electrocardiogram with a non-standard lead. The following hemodynamic parameters were measured either directly or calculated, or referenced to body surface area (BSA), using original software: heart rate (HR), stroke volume (SV), cardiac output (CO), cardiac index (CI), systemic vascular resistance index (SVRI) and total arterial compliance index (TACI). 

Additionally, by using the same ICG device and appropriate upper arm cuff, oscillometric measurement of the blood pressure was performed, which allowed repeated measurement of the SBP, DBP and MABP values. 

### 4.6. Peripheral Microvascular Response to Local Thermal Hyperemia (LTH) 

Peripheral microvascular response to local thermal hyperemia (LTH) using laser Doppler flowmetry (LDF) was used to assess specifically NO-mediated endothelial-dependent vasodilatation of the skin microcirculation. Local heating of the skin is known to produce a biphasic rise in skin blood flow; a rapid initial increase within the first 90–120 s following the onset of heating, followed by a prolonged rise in blood flow which reaches a plateau after 20–30 min of heating. An initial peak appears to be predominantly the result of a local sensory nerve axon reflex, while the plateau phase appears to be predominantly mediated by endothelial factors, more specifically by NO (60–70% of the plateau response) [52]. LDF measurement was performed at each study visit in a temperature-controlled room (mean ± SD temperature = 23.5 ± 0.5 °C). The laser Doppler probe was attached to the skin of the volar forearm, 13–15 cm from the wrist, at the same place at each study visit. Skin microvascular flow was measured during a 5 min baseline, using a laser Doppler monitor (moorVMS-LDF, Moor instruments, Axminster, UK). After baseline measurements, the local temperature was increased from baseline level to 42 °C at a rate of 0.1 °C s^−1^ and remained at 42 °C for the duration of the heating protocol, using a skin heater controller (moorVMS-HEAT, Moor instruments, Axminster, UK). Blood flow was measured during the entire LTH protocol, until blood flow reached a stable plateau (30–45 min minutes following the start of heating). Microcirculatory blood flow was expressed in arbitrary perfusion units and determined by software calculating the area under the curve (AUC) during the baseline flow and stable heating plateau. The result was expressed as the flow increase during heating compared to the baseline microvascular flow.

### 4.7. Flow-Mediated Dilation (FMD) of Brachial Artery 

Brachial artery flow-mediated dilation (FMD) is a technique commonly used in clinical research to measure arterial function in vivo as percentage dilation of the brachial artery after a period of forearm occlusion. Moreover, FMD presents the current gold standard which quantifies NO-dependent arterial vasodilation [53,54]. Imaging of the brachial artery was performed in a longitudinal plane, at approximately 5 cm proximal to the antecubital fossa of the right arm, abducted approximately 80° from the body, with the forearm supinated. The vascular linear ultrasound probe (Vivid™ iq, GE Health Care, Chicago, IL, USA) was positioned at a 60° insonation angle to visualize the anterior and posterior lumen–intima interfaces, to measure diameter or central flow velocity (pulsed Doppler). After baseline, Doppler readings of flow velocity were performed. A blood pressure cuff was placed on the forearm, distal to the antecubital fossa of the imaged arm, and inflated to 60 mmHg above baseline SBP for 5 min. The brachial artery was then imaged continuously after BP cuff release. The response to NTG was used for the determination of endothelium-independent vasodilation. One sublingual NTG spray push (0.4 mg) was administered after obtaining a baseline brachial diameter, and brachial artery images and measurements were repeated for 5 min. Images were digitally recorded using Brachial Imagery (Medical Imaging Applications, Iowa City, IA, USA) and analyzed as previously described [9]. FMD and the response to NTG were calculated, using the average with the largest mean values obtained after release of the forearm occlusion or administration of NTG.

### 4.8. Serum Nitric Oxide (NO) and Three Isoforms of Nitric Oxide Synthase (NOS) Assay

Serum concentration of NO, as well as endothelial (eNOS), inducible (iNOS) and neuronal (nNOS) nitric oxide synthase was measured on a compact absorbance reader for 96-well microplates using commercially available enzyme-linked immunosorbent assay (ELISA) kits (Cusabio Technology LLC, Houston, TX, USA), according to the manufacturer’s instructions.

### 4.9. Serum Vascular Endothelial Growth Factor C (VEGF-C) Assay

Serum vascular endothelial growth factor C (VEGF-C) concentration was measured on a compact absorbance reader for 96-well microplates using a commercially available enzyme-linked immunosorbent assay (ELISA) kit (Abbexa Ltd., Milton, Cambridge, UK), according to the manufacturer’s instructions.

### 4.10. Statistical Analysis

The results were presented using the arithmetic mean and standard deviation (SD) for the normally distributed data, while non-normally distributed data were presented using the median and the min.-to-max. range. The normality of the distribution of numerical variables were determined by the Kolmogorov–Smirnov normality test. Differences of normally distributed variables between measurements before and after the HS diet (LS vs. HS condition) were tested with a *t*-test for dependent samples (paired *t*-test), and in the case of deviation from a normal distribution, with the Wilcoxon rank sum test. The association (correlation) between the corresponding variables was determined by Pearson’s or Spearman’s correlation tests, depending on the normality of the data distribution. A two-sided *p* ≤ 0.05 was considered statistically significant. The statistical program SigmaPlot (SYSTAT Software version 11.2, Chicago, IL, USA) was used for statistical analysis.

## Figures and Tables

**Table 1:** Effects of a 7-Day High-Salt Diet on Weight Status, Body Composition and Body Fluid Status in Young Healthy Individuals.

Parameter	LS	HS
N (W/M)	46 (24/22)	
Age (years)	21 [18–24]	
*Weight Status*		
BMI (kg/m^2^)	23.1 [17.8–29.9]	23.1 [17.8–29.9]
Waist Circumference (cm)	77.8 ± 9.4	77.6 ± 9.3
*Body Composition and Fluid Status*		
Fat Free Mass (%)	82.0 ± 5.7	82.3 ± 5.4
Fat (%)	18.0 ± 5.7	17.7 ± 5.4
Total Body Water (%)	61.4 ± 6.3	61.4 ± 4.9
Extracellular Water (%)	42.9 ± 1.4	42.8 ± 1.2
Intracellular Water (%)	57.1 ± 1.4	57.2 ± 1.2
ECW/ICW	0.75 ± 0.04	0.75 ± 0.04
Plasma Fluid (L)	3.8 ± 0.8	3.8 ± 0.9
Interstitial Fluid (L)	13.4 ± 2.8	13.4 ± 3.2
Body Density (kg/L)	1.06 ± 0.01	1.06 ± 0.01

Data are presented as the mean ± standard deviation (SD) (normally distributed data) or as the median and min-to-max range (non-normally distributed data). LS—low salt; HS—high-salt; N—number of participants; W—women; M—men; BMI—body mass index; ECW—extracellular water; ICW—intracellular water.

**Table 2:** Effects of a 7-Day High-Salt Diet on Standard Biochemical Venous Blood and 24-Hour Urine Parameters in Healthy Individuals.

Parameter	LS	HS	Reference Range ^†^
N (W/M)	46 (24/22)		
*Serum Biochemical Parameters*			
urea (mmol/L)	5.2 ± 1.4	5.5 ± 1.3	2.8–8.3
creatinine (µmol/L)	78.3 ± 12.7	76.9 ± 12.3	64–104
sodium (mmol/L)	139.0 ± 12.7	138.9 ± 12.3	137–146
potassium (mmol/L)	4.3 ± 0.4	4.2 ± 0.2	3.9–5.1
calcium (mmol/L)	2.46 ± 0.08	2.43 ± 0.07 *	2.14–2.53
hsCRP (mg/L)	0.84 [0.20–4.09]	0.64 [0.12–1.70]	<5.00
*24-h Urine Biochemical Parameters*			
24 h urine volume (mL)	1560 ± 714	1826 ± 723 *	
24 h creatinine coefficient (µmol/24 h/kg)	182 ± 45	191 ± 50	94–253
24 h urine urea (mmol/dU)	333 ± 122	364 ± 140	200–350
24 h urine protein (mg/dU)	88 [23–298]	108 [27–183]	<150
24 h urine albumin (mg/dU)	9.83 ± 10.88	9.17 ± 8.18	<30.0
24 h sodium (mmol/dU)	118 ± 42	267 ± 82 *	40.0–220.0
24 h potassium (mmol/dU)	50.4 [11.5–121.2]	58.7 [22.7–132.6]	25.0–125.0
calculated salt intake (g/day)	6.7 ± 2.6	15.6 ± 4.8 *	

Data are presented as the mean ± standard deviation (SD) (normally distributed data) or as the median and min-to-max range (non-normally distributed data). LS—low salt; HS—high-salt; N—number of participants; W—women; M—men; hsCRP—high-sensitivity C reactive protein. * *p* < 0.05 LS vs. HS (Paired *t*-test). ^†^ Source: Department of Clinical Laboratory Diagnostics, University Hospital Centre Osijek, Osijek, Croatia.

**Table 3:** Hemodynamic Responses to a 7-Day High-Salt Diet in Young, Healthy Individuals.

Parameter	LS	HS
N (W/M)	46 (24/22)	
*Hemodynamic Parameters*		
Systolic ABP (mmHg)	115 ± 10	115 ± 12
Diastolic ABP (mmHg)	74 ± 8	72 ± 10
Mean ABP (mmHg)	87 ± 7	86 ± 9
Heart Rate (beats/min)	74 ± 13	73 ± 9
Stroke Volume (mL)	96.7 ± 26.7	93.0 ± 17.0
Cardiac Output (L/min)	6.96 ± 1.30	6.72 ± 1.12
Cardiac Index (L/min/m^2^)	3.86 ± 0.54	3.71 ± 0.48
Systemic Vascular Resistance Index (dyn.s.cm^−5^ m^2^)	1639 ± 297	1754 ± 318
Total Arterial Compliance Index (mL/m^2^/mmHg)	1.087 ± 0.269	1.056 ± 0.193

Data are presented as the mean ± standard deviation (SD) (normally distributed data) or as the median and interquartile range (non-normally distributed data). LS—low salt; HS—high-salt; N—number of participants; W—women; M—men; ABP—arterial blood pressure. LS vs. HS (Paired *t*-test).

**Table 4:** Effects of a 7-Day High-Salt Diet on Nitric Oxide and Vascular Endothelial Growth Factor C (VEGF-C) Serum Levels in Young, Healthy Individuals.

Parameter	LS	HS
N (W/M)	46 (24/22)	
*Nitric Oxide Serum Level*		
NO (μmol/L)	39.5 ± 8.3	39.5 ± 11.0
*VEGF-C Serum Level*		
VEGF-C (ng/mL)	2.93 ± 0.90	2.78 ± 0.69

Data are presented as the mean ± standard deviation (SD) (normally distributed data) or as the median and interquartile range (non-normally distributed data). LS—low salt; HS—high salt; N—number of subjects; W—women; M—men; NO—nitric oxide. LS vs. HS (Paired *t*-test).

**Table 5:** Correlations between 24-h sodium excretion/NO serum concentration and endothelial vascular response/NOS isoforms serum concentrations.

		24-h Sodium Excretion (mmol/dU)	NO Serum Concentration (μmol/L)
LTH (flow increase)	R	−0.254	0.555
	*p*	0.050 *	0.017 *
FMD (% of dilation)	R	−0.322	−0.268
	*p*	0.015 *	0.278
eNOS (IU/mL)	R	0.071	−0.128
	*p*	0.664	0.648
nNOS (IU/mL)	R	−0.483	−0.166
	*p*	0.002 *	0.562
iNOS (IU/mL)	R	0.199	0.267
	*p*	0.224	0.348

R- correlation coefficient; *p*—*p* value; NO—nitric oxide; NOS—nitric oxide synthase; LTH—local thermal hyperemia; FMD—flow-mediated dilation; eNOS—endothelial nitric oxide synthase; nNOS—neuronal nitric oxide synthase; iNOS—inducible nitric oxide synthase. * *p* < 0.05, Pearson’s or Spearman’s correlation tests (depending on the normality of the data distribution).

## Data Availability

The data presented in this study are available on request from the corresponding authors.
